# Effectiveness of mHealth interventions for maternal, newborn and child health in low– and middle–income countries: Systematic review and meta–analysis

**DOI:** 10.7189/jogh.06.010401

**Published:** 2016-06

**Authors:** Siew Hwa Lee, Ulugbek B Nurmatov, Bright I Nwaru, Mome Mukherjee, Liz Grant, Claudia Pagliari

**Affiliations:** 1 eHealth Research Group, Usher Institute of Population Health Sciences and Informatics, The University of Edinburgh Medical School, Edinburgh, UK; 2 Edinburgh Health Services Research Unit, Edinburgh, UK; 3 Edinburgh Global Health Academy, The University of Edinburgh Medical School, Edinburgh, UK

## Abstract

**Objective:**

To assess the effectiveness of mHealth interventions for maternal, newborn and child health (MNCH) in low– and middle–income countries (LMIC).

**Methods:**

16 online international databases were searched to identify studies evaluating the impact of mHealth interventions on MNCH outcomes in LMIC, between January 1990 and May 2014. Comparable studies were included in a random–effects meta–analysis.

**Findings:**

Of 8593 unique references screened after de–duplication, 15 research articles and two conference abstracts met inclusion criteria, including 12 intervention and three observational studies. Only two studies were graded at low risk of bias. Only one study demonstrated an improvement in morbidity or mortality, specifically decreased risk of perinatal death in children of mothers who received SMS support during pregnancy, compared with routine prenatal care. Meta–analysis of three studies on infant feeding showed that prenatal interventions using SMS/cell phone (vs routine care) improved rates of breastfeeding (BF) within one hour after birth (odds ratio (OR) 2.01, 95% confidence interval (CI) 1.27–2.75, I^2^ = 80.9%) and exclusive BF for three/four months (OR 1.88, 95% CI 1.26–2.50, I^2^ = 52.8%) and for six months (OR 2.57, 95% CI 1.46–3.68, I^2^ = 0.0%). Included studies encompassed interventions designed for health information delivery (n = 6); reminders (n = 3); communication (n = 2); data collection (n = 2); test result turnaround (n = 2); peer group support (n = 2) and psychological intervention (n = 1).

**Conclusions:**

Most studies of mHealth for MNCH in LMIC are of poor methodological quality and few have evaluated impacts on patient outcomes. Improvements in intermediate outcomes have nevertheless been reported in many studies and there is modest evidence that interventions delivered via SMS messaging can improve infant feeding. Ambiguous descriptions of interventions and their mechanisms of impact present difficulties for interpretation and replication. Rigorous studies with potential to offer clearer evidence are underway.

Mortality in children under the age of five has fallen from an average rate of 90 per 1000 live births in 1990 to 43 in 2015, while maternal mortality has declined by 45% [1]. Despite these improvements, progress in achieving Millennium Development Goals 4 and 5 fell short of expectations, and low– and middle–income countries (LMIC) account for 97% of maternal and 94% of neonatal mortality [2,3]. The availability and quality of maternal health care varies widely in different parts of the world and in LMIC women continue to die each year from preventable causes [4–6]. This is further compounded by limited resources and poor information infrastructures, which act as barriers to care coordination and quality, and hinder the effective management and governance of health systems [7–11].

mHealth, or mobile health, refers to the use of wireless, portable Information and Communication Technologies (ICT) to support health and health care [12]. There are numerous examples of mHealth interventions being used to support mothers through safe pregnancy and childbirth and to facilitate neonatal and infant health. Although scaled programmes do exist, the majority of mHealth projects in LMIC have tended to be small–scale donor–funded initiatives, which have taken place without the benefit of an adequate evidence–base [13].

A number of efforts have attempted to map the state of the evidence relating to mHealth for maternal, newborn and child health (MNCH) in LMIC, but no rigorous systematic reviews exist on this specific topic [14–16]. Philbrick’s ‘gap analysis’, for the mHealth Alliance, combined literature review and stakeholder interviews [17], whilst literature reviews by Noordam et al. and Tamrat and Kachowski addressed the topic using simple search terms and a subset of available databases [18,19]. Free et al. reported two broader systematic reviews of interventions for patient behavior change and for health care service delivery processes and, while studies from LMIC were not excluded, the focus was higher income country settings [20,21]. In another mHealth report, Labrique et al. reviewed existing research for the purposes of developing a taxonomy of interventions [22]. While all of these provided valuable insights and recommendations, the World Health Organization (WHO) recognised the need for a rigorous systematic review when commissioning the current study. As we move on from the Millennium Development Goals and plan forward strategies for improving MNCH, mHealth is likely to play an increasing important role in light of continuing health needs and the growing global penetration of mobile technologies.

This study synthesized the evidence on the effect of mHealth interventions on MNCH in LMIC, with a particular focus on studies reporting impacts on patient outcomes.

## METHODS

A detailed protocol was registered with the International Prospective Register for Systematic Reviews (PROSPERO) CRD42014008939 (http:///www.crd.yourk.ac.uk/prospero) and has been published [23]. The review is reported according to the requirements of the Preferred Reporting Items for Systematic Reviews and Meta–Analyses (PRISMA) [24]. We assessed studies that have investigated the effectiveness of mHealth interventions for improving MNCH in LMIC. LMIC were identified in accordance with World Bank country classifications [25]. The target groups were women in the antenatal, intranatal, and postnatal periods; newborns; children aged 0–5 years; and health workers through which interventions aimed at these groups are mediated. Men, non–pregnant women or those not recently having given birth, and children over the age of 5 years were excluded. We included studies evaluating interventions delivered through mobile ICT and considered the various delivery modes through which this might be achieved (**Box 1**). We excluded related ICT–based interventions delivered via fixed line internet or standard telephone line, interventions labeled ‘mobile’ which did not involve cellphones, such as Mobile Maternal Health Clinics which are touring buses staffed by health care professionals.

Box 1 **Mobile ICT and delivery modes**

Mobile ICT includes: cell–phones, smart–phones, satellite phones, personal digital assistants, enterprise digital assistants, tablet computers, laptops, portable media players and gaming consoles, Radio Frequency Identification Device (RFID) tags, Global Positioning System (GPS) trackers and digital diagnostic devices.

Mobile delivery modes includes: voice calling, Voice over Internet Protocol (VoIP), text messaging via Short Message Service (SMS), transfer of still or moving images via Multimedia Message Service (MMS), multimedia downloads, and live video.

The primary outcomes were estimates of maternal, newborn and child mortality and morbidity. Secondary outcomes included number of planned antenatal and postnatal visits; number of unscheduled care visits and emergency care; quality of life; quality of care (delivery by skilled birth attendants, appropriate use of evidence–based medical and obstetric interventions); self–efficacy; cost–effectiveness; immunisation cover; child developmental milestones; and other process indicators.

### Search strategy and study selection

16 international electronic databases were interrogated (**Box 2**) using highly sensitive search strategies implemented in OVID MEDLINE and then adapted to other databases (see Tables s1 and s2 in **Online Supplementary Document(Online Supplementary Document)**). Searches were limited to articles published between January 1990 and May 2014, acknowledging the emergence of digital cellular networks in the early 1990s [14]. The search strategies were piloted in order to optimise sensitivity and specificity. The decision was taken to dispense with country restrictions after finding that limiting searches to the LMIC countries specified in the World Bank’s classification scheme had resulted in the omission of a highly relevant study from Zanzibar. (Although Zanzibar is part of Tanzania, which is listed, the word Tanzania did not appear in the title or abstract, hence the article was ignored.) There were no restrictions on language of publication. We included randomised controlled trials (RCTs), variations of RCTs, controlled before and after studies, interrupted time series studies and observational studies (cohort, case–control). We excluded cross–sectional and qualitative studies, expert opinions, reports, discussion papers, case reports, and studies from developed countries. Authors were contacted for access to unpublished research.

Box 2 **Sources of literature included in this systematic review and meta–analyses**

**Databases:**

• Cochrane Library (Cochrane Database of Systematic Reviews, Cochrane Central Register of Controlled Trials (CENTRAL), Cochrane Methodology Register),

• MEDLINE

• EMBASE

• CINAHL

• PsycINFO

• AMED

• Global Health

• TRIP

• ISI Web of Science (Science and Social Science Index)

• WHO Global Health Library

• IndMed

• PakMediNet

• KoreaMed

• NHS Health Technology Assessment Database

• African Index Medicus (encompassed in the WHO Global Health Library)

• POPLINE

**Clinical trials registry for on–going studies and trial protocols:**

• WHO International Clinical Trials Registry platform

• Clinical trials.gov

• Controlled–trials.com

• Australian New Zealand Clinical Trials Registry

**Reference tracking:**

• References list of all included studies

At least two reviewers independently screened the titles and abstracts of identified studies, assessed the full text of potentially eligible studies against the inclusion and exclusion criteria, and abstracted relevant study data onto a customised data extraction sheet. Country classification was undertaken by hand. Due to the large number of articles, and annual fluctuations in the World Bank index, a pragmatic decision was taken to include countries classed as LMIC at any time during the search period, or otherwise described using a phrase such as “developing country” (as described in the protocol).

### Assessment of risk of bias

The methodological quality of intervention studies was assessed independently by at least two reviewers, following the recommendations of the Cochrane Effective Practice and Organization of Care Group [26]. Observational studies were assessed using the Effective Public Health Practice Project quality assessment tool [27]. Discrepancies were resolved by team consensus.

### Meta–analysis

There was substantial heterogeneity between studies with regards to the mHealth interventions and study outcomes, except for the studies on breastfeeding (BF) and infant feeding [28–44]. Consequently, we performed a random–effects meta–analysis using the inverse variance method for three comparable studies, which had all used SMS/cell phone as the intervention vs routine prenatal care and had assessed breastfeeding as the primary outcome [30,33,42]. The study by Sellen et al. compared cell phone–based peer support, monthly peer support group and standard existing routine care for BF [42]. However in the meta–analysis we compared only the cell phone group with the routine care group as the relevant intervention for the review. The estimates of effect in the study by Sellen et al. were given as percentages [42], but we recalculated these into odds ratios with their 95% confidence intervals (95% CI) before the pooled analysis. Given the small number of studies in each meta–analysis, we did not explore reasons for the observed heterogeneity. For the same reason, we did not investigate the influence of publication bias or undertake possible sensitivity analyses. Meta–analyses were undertaken using STATA 11 (Stata Corp, College Station, Tx) [45].

## RESULTS

### Study selection and characteristics

**Figure 1:**
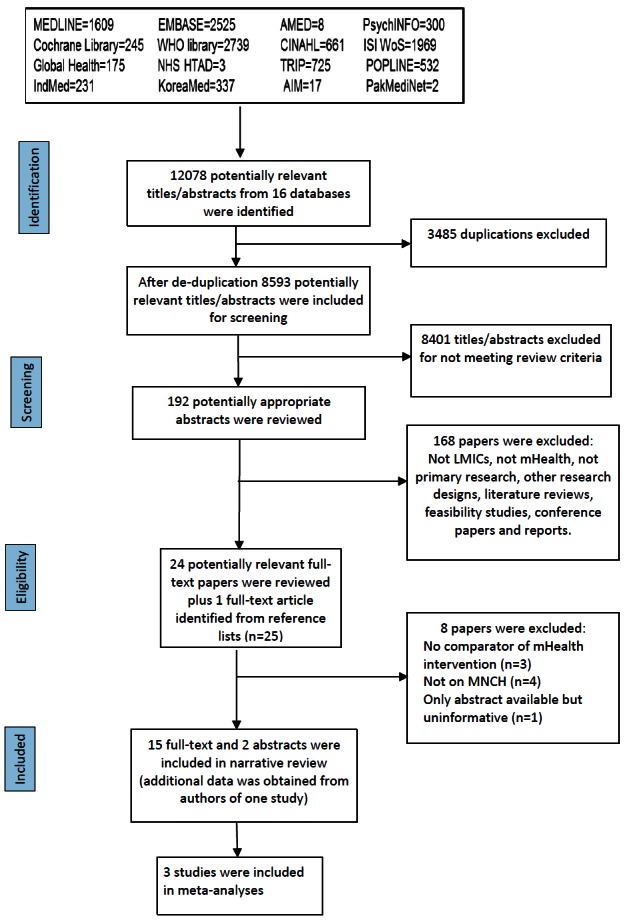
PRISMA flow diagram for database search of studies on mHealth interventions for maternal, newborn and child health in low– and middle–income countries, 1990–2014.

Initial searches identified 12078 titles. After removing duplicates, 8593 papers were included for initial screening. Of these, 8401 papers were excluded after screening by title and abstract, leaving 192 papers, which were considered in more detail. A further 168 papers were subsequently excluded for not meeting the relevant criteria. 24 papers remained, and one additional paper was identified through searching the reference lists of these papers. Of the 25 full–text papers, 17 met the inclusion criteria and were included in the final review (**Figure 1**). These were based on 15 primary studies [28-34,36–41,43,44], of which two were only available as conference abstracts [35,42].

Twelve of the eligible studies were intervention studies, comprising eight RCTs [28,30,32,34,36,37–39,42,43], two quasi–RCTs [33,44], one controlled clinical trial (CCT) [29], and one uncontrolled before and after study [41]. Two studies were cohort studies [31,35] and one was a case–control study (**Table 1**) [40]. Seven studies were undertaken in Sub–Saharan Africa (Kenya [31,42], Mali [44], Nigeria [30,40], Tanzania [37–39], and Zambia [41]), five in East Asia (China [33,36], Taiwan [28,29], and Thailand [32]), two in South Asia (Bangladesh and India) [35,43] and one in the Middle East (Iran) [34]. All the studies were published between 2008 and 2014. The study population comprised pregnant women in ten studies [28,29,30,32,33,34,35,37–39,40, 42], children in five studies [31,36,41,43,44], and village elders in one (**Table 1**) [31].

**Table 1:** Characteristics and results of studies investigating the effectiveness of mHealth interventions for maternal, newborn and child health in low– and middle– income countries during January 1990 – May 2014

Study and country	Study design and setting	Study population	Intervention/Exposure	Outcomes	Results	Overall risk of bias grading	Classification of interventions
Cheng et al. (2008), Taiwan [28]	Randomised controlled trial (RCT), Hospital	Pregnant women at 14–18 weeks of gestation. Total N = 2782 Intervention group = 1422 Control Group = 1360	Report of results of Down Syndrome via SMS vs report at the time of routine clinic appointment	*Primary outcomes:* Anxiety levels of women as measured by Trait–and Stat–anxiety scores	*Negative results for Down Syndrome:* Trait–anxiety score (*P* = 0.69): SMS group mean 39.8 ± 11.2; Control group mean 38.4 ± 10.9 State–anxiety score Before screening (*P* = 0.51): SMS group mean 38.9 ± 9.9; Control group mean 37.8 ± 11.3 After screening (*P* = 0.02): SMS group mean 33.8 ± 7.9; Control group mean 39.1 + 10.1 *Positive results for Down Syndrome:* Trait–anxiety score (*P* = 0.57): SMS group mean 38.7 ± 8.8; Control group mean 40.1 ± 13.2 State–anxiety score Before screening (*P* = 0.66): SMS group mean 39.2 ± 11.4; Control group mean 39.9 ± 9.4 After screening (*P* = 0.21): SMS group mean 44.1 ± 13.4; Control group mean 42.9 ± 11.5	High	Test result turnaround
Chuang et al. (2012), Taiwan [29]	Controlled Clinical Trial, Hospital	Women diagnosed with preterm labour at 20–34 weeks of gestation Total N = 129. Intervention group = 68 Control group = 61	13–minute relaxation audio program via mp3 player vs no mp3 player (routine prenatal care)	*Primary outcomes:* Gestation at birth; new–born birth weight; Apgar score; perinatal mortality; admission to neonatal intensive care unit; number of days of prolongation of pregnancy	*Gestational weeks at birth (P = 0.217):* Mp3 player group mean 35.2 ± 4.4; Control group mean 34.2 ± 4.5 *Birth weight in grams (P = 0.296):* Mp3 player group mean 2389.2 ± 828 Control group mean 2266.6 ± 898 *Apgar score at 1 min (P = 0.782):* Mp3 player group mean 7.9 ± 2.0 Control group mean 7.8 ± 2.0 *Apgar score at 5 min (P = 0.732):* Mp3 player group mean 9.2 ± 1.9 Control group mean 9.0 ± 1.9 *Route of delivery (P = 0.918):* Normal: mp3 player group 52.9%; control group 54.2% Caesarean section: mp3 player group 47.1% control group 45.8% *Perinatal mortality (P = 0.337):* Mp3 player group 1.5%; control group 5.1%	Moderate	Psychological (therapeutic) intervention – Tailored exercises (audio recordings)
Flax et al. (2014), Nigeria [30]	Cluster RCT, General population	Pregnant women aged between 15–45 y. Total N = 461 Intervention group = 229 Control group = 232	Breastfeeding (BF) learning sessions and SMS and songs/dramas vs none of these (routine care)	*Primary outcomes:* –Exclusive BF to 1, 3, and 6 months –Initiation of BF within 1 h of delivery –Use of colostrum or breast milk within the first 3 d of life.	*Exclusive BF at 1 months:* Intervention group 73%; Control group 61%; OR 1.6 (95% CI 0.6–1.8) *Exclusive BF at 3 months:* Intervention group 71%; Control group 58%; OR 1.8 (95% CI 1.1–3.0) *Exclusive BF at 6 months:* Intervention group 64%; Control group 43%; OR 2.4 (95% CI 1.4–4.0) *Initiated BF within 1 h of delivery:* Intervention group 70%; Control group 48%; OR 2.6 (95% CI 1.6–4.1) *Gave only colostrum/breast milk during the first 3 d:* Intervention group 86%, Control group 71%; OR 2.6 (95% CI 1.4–5.0)	Moderate	Health Information delivery – Education messages sent to group leaders as part of complex change intervention (SMS+Voice messaging) – Group–mediated socio–cultural intervention (SMS±Voice Messaging)
Gisore et al. (2012), Kenya [31]	Cohort study General population	Village elders Total N = 474	Use of mobiles by village elders for pregnancy case finding and reporting birth weights	*Primary outcomes:* –% change in birth weights reported by mobile phones compared to previous national estimates –% of women enrolled after delivery	Recorded birth weights increased from 43 ± 5.7% to 97 ± 1.1% % of women enrolled after delivery decreased from 30.4% to 25%, *P* < 0.0001	High	Data collection (health monitoring or case finding by Community Health Workers)
Jareethum et al. (2008), Thailand [32]	RCT Hospital	Pregnant women at <28 weeks gestation Total N = 61 Intervention group = 32 Control group = 29	SMS via mobile phone for prenatal support vs no SMS (routine prenatal care)	*Primary outcome:* Mothers’ level of satisfaction with antenatal care *Secondary outcomes:* –Mothers’ confidence level at prenatal care –Mothers’ anxiety level at prenatal care –Gestational weeks at delivery –Foetal birth weight –Route of delivery –Preterm delivery	*Mothers’ level of satisfaction with prenatal care (P = <0.001):* SMS group mean 9.3 ± 0.7; Control group mean 8.0 ± 1.1 *Mothers’ confidence level at prenatal care (P = 0.001):* SMS group mean 8.9 ± 0.9; Control group mean 7.8 ± 1.5 *Mothers’ anxiety level at prenatal care (P = 0.002):* SMS group mean 2.8 ± 2.1; Control group mean 4.9 ± 2.9 *Gestational weeks at delivery (P = 0.340):* SMS group mean 38.7 ± 1.1; Control group mean 38.6 ± 1.1 *Foetal birth weight in grams (P = 0.350):* SMS group mean 3051 ± 636; Control group mean 3188 ± 456 *Preterm delivery (P = 0.220):* SMS group 0%; Control group 6.9% *Route of delivery (P = 1.00):* Normal vaginal delivery: SMS group 81.3%; Control group82.8% Caesarean section: SMS group18.7%; Control group17.2%	Moderate	Health Information Delivery – Tailored information (also labelled ‘advice’ and ‘support’) (SMS)
Jiang et al. (2014), China [33]	Quasi–RCT Community Health Centres	Pregnant women at <13 weeks gestation Total N = 582 Intervention group = 281 Control group = 301	Text via SMS vs no SMS (routine prenatal care)	*Primary outcome:* Duration of exclusive BF *Secondary outcomes:* –Rate of exclusive BF at 6 months –Duration of any BF –Timing of intro. solid foods –Rate of BF at 12 months –Rates of other infant feeding behaviours	*Exclusive BF at 4 months:* SMS group 46.4%; Control group 39.9%; OR 1.4 (95% CI 1.0–2.0) *Exclusive BF at 6 months:* SMS group 15.1%; Control group 6.3%; OR 2.7 (95% CI 1.5–4.9) *BF at 12 months:* SMS group 20.2%; Control group 19.2%; OR 1.0 (95% CI 0.7–1.6) *Introduction of solid foods before 4 months:* SMS group 1.5%; Control group 3.8%; OR 0.3 (95% CI 0.1–0.9) *Introduction of solid foods before 6 months:* SMS group 67.5%; Control group 61.3%; OR 1.3 (95% CI 0.9–1.8) *Drinking from a cup at 12 months:* SMS group 53.6%; Control group 46.5%; OR 1.3 (95% CI 0.9–2.0) *Receiving food as a reward:* SMS group 45.5%; Control group 33.6%; OR 1.5 (95% CI 1.0–2.3) *Taking a bottle to bed:* SMS group 51.9%; Control group 49.8%; OR 1.1 (95% CI 0.7–1.6)	High	Health Information Delivery – Tailored information/promotion (also labelled ‘education’ and ‘support’) (SMS)
Khorshid et al. (2014), Iran [34]	RCT Public Health Centres	Pregnant women at gestational 14–16 weeks Total N = 116 Intervention group = 58 Control group = 58	A 12–week SMS reminders in addition to usual care vs no SMS reminders (only usual care) on compliance with intake of iron supplements	*Primary outcome:* Compliance with intake of iron supplements *Secondary outcomes:* Measures of blood indices for anaemia (haemoglobin, haematocrit, ferritin)	*Compliance with intake of iron supplements (P = 0.003):* High compliance: SMS group 94%; Control group 66% Moderate compliance: SMS group 4%; Control group 18% Low compliance: SMS group 2%; Control group 16% *Measures of blood indices for anaemia:* Haemoglobin in g/dL (*P* = 0.960): SMS group mean 11.2 ± 0.5; control group mean 11.2 ± 0.9 Haematocrit, % (*P* = 0.670): SMS group mean 33.9 ± 1.7; control group mean 34.0 ± 2.6 Ferritin in ng/dL (*P* = 0.630): SMS group mean 24.4 ± 35.0; control group mean 22.5 ± 19.7	Moderate	Health Information Delivery – Health ‘education’ (SMS)
Labrique et al. (2011), Bangladesh [35]*	Follow–up analysis of RCT General population	Pregnant women interviewed at 1 month postpartum to collect information on complications of labour and delivery Total *N*>100 000)	Use of mobile phones to report obstetric emergencies	*Primary outcomes:* –Reported use of mobile phones during intrapartum	55.2% of women reported using a mobile phone for obstetric emergencies. Of these: 57.0% to receive medical advice 71.7% to call a health care provider 32.6% to arrange for transportation 20.9% to ask for financial support.	N/A	Communication Platform (one way or two way interpersonal communication) – Patient with Health Care Providers (Voice)
Lin et al. (2012), China [36]	RCT Hospital	Parents of children with diagnosis of cataract aged <18 years Total N = 258. Intervention group = 135 Control group = 123	Text messaging via SMS vs standard follow–up appointments	*Primary outcome:* Rate of attendance at scheduled study appointments *Secondary outcomes:* –Additional procedures (surgeries, laser treatments for posterior capsular opacification, or changes in eyeglass prescription) –Occurrence of secondary ocular hypertension	*Attendance rates (P = 0.003):* SMS group 91.3%; Control group 62.0%; RR: 1.47 (95% CI 1.16–1.78) *Secondary outcomes:* Surgeries (*P* = 0.03): SMS group 43.0% ; Control group 27.6%) ; RR 1.55 (95% CI 1.10–2.20) Laser for capsular opacification (*P* = 0.008): SMS group 46.0%; Control group 18.7%; RR 2.46 (95% CI 1.63–3.71) Prescription of new glasses (*P* = <0.001): SMS group 71.1%; Control group 52.8%; RR 1.35 (95% CI 1.10–1.64) Treatment for ocular hypertension (*P* = 0.04): SMS group 23.0%; Control group 9.8%; RR 2.35 (95% CI 1.27–4.38)	Low	Reminders (Cognitive) – Personalised, appointment (SMS)
Lund et al. 2012, 2014a, 2014b, Zanzibar, Tanzania [37–39]	Pragmatic Cluster–RCT General population	Pregnant women at first prenatal care attendance Total N = 2637 Intervention group = 1351 Control group = 1286	Mobile phone vouchers and SMS vs no mobile phones (routine care)	*Primary outcomes:* –Skilled delivery attendance –Number of women receiving four or more antenatal care visits *Secondary outcomes:* –Home delivery assisted by skilled birth attendants –Quality of care in terms of content and timing of antenatal care **–**Stillbirth –Perinatal death –Death of a child within 42 d of life	*Skilled delivery attendance:* SMS group 60%; Control group 47% *Four or more antenatal visits:* SMS group 44%; Control group 31%; a OR 2.39 (95% CI 1.03–5.55) *Secondary outcomes:* **–**Tetanus toxoid vaccination at first antenatal care visit: SMS group 96%; Control group 94%; aOR 1.58 (95% CI 0.41–6.01) –Tetanus toxoid vaccination at least 4 weeks after first antenatal care visit: SMS group 72%; Control group 56%; aOR 1.62 (95% CI 0.81–3.26) –Intermittent preventive treatment in pregnancy at first prenatal visit: SMS group 91%; Control group 86%; aOR 1.10 (95% CI 0.35–3.43) –Intermittent preventive treatment in pregnancy at least 4 weeks after first prenatal visit: SMS group 65%; Control group 52%; aOR 1.97 (95% CI 0.98–39.4) –Gestational age 36 or more at last antenatal care visit: SMS group 28%; Control group 20%; aOR 1.48 (95% CI 0.89–2.45) –Antepartum referral: SMS group 10%; Control group 5%; aOR 1.66 (95% CI 0.68–4.06) –Stillbirth: SMS group 17 per 1000 births; Control group 26 per 1000 births; aOR 0.65 (95% CI 0.34–1.24) –Perinatal mortality: SMS group 19 per 1000 births; Control group 36 per 1000 births; aOR 0.50 (95% CI 0.27–0.93) –Death of child <42 d after birth: SMS group 14 per 1000 births; Control group 15 per 1000 births; aOR 0.79 (95% CI 0.36–1.74)	Moderate	Health Information Delivery – Tailored education (SMS) – Reminders (Cognitive) – Personalised, appointment (SMS)
Oyeyemi and Wynn (2014), Nigeria [40]	Case–control study General Population	Pregnant women Cases = 1429 Controls = 1801	Giving mobile phones to pregnant women to increase primary health facility utilisation (cases) vs no mobile phones (controls)	*Primary outcome:* Facility utilisation rate *Secondary outcome:* Frequency of occurrence of 5 major causes of maternal deaths (severe bleeding, hypertensive disorder of pregnancy with fits, infection, obstructed labour, unsafe abortion)	*Facility utilisation:* Cases 43.4%; Controls 36.6; OR 1.32 (95% CI 1.15–1.53) *Number of illness cases:* Cases 1.6%; Controls 1.6%; OR 1.00, (95% CI 0.58–1.74)	Moderate	Communication Platform – One– or two–way interpersonal communication (Voice)
Seidenberg et al. (2012), Zambia [41]	Before and after study General population	All infants who came for antenatal care Before program = 1009 After program = 406	Notification of blood results of infant diagnosis of HIV infection through SMS vs postal notification	*Primary outcomes:* –Mean turnaround time (time from sample collection to delivery of test result to either the relevant point–of–care health facility or a caregiver of the tested infant) –Result error rate (per cent discordance between the results recorded on paper and the corresponding results sent by SMS)	*Turnaround (days) at relevant health facility:* Before program: mean 44.2 ± 28.0 After program: mean 26.7 ± 31.8 Difference in mean days: –17.5 (95% CI –14.1 to –20.9) *Turnaround (days) to a caregiver:* Before program: mean 68.8 ± 38.8 After program: 35.0 ± 31.2 Difference in mean: –33.8 (95% CI –28.7 to –38.9) *Per cent discordance:* Number of samples agreed by paper and SMS = 336 Number of discrepancies = 2 Error rate 0.5%	Moderate	Test result turnaround – To facility
Sellen et al. (2013), Kenya, [42]*	RCT Hospital	Pregnant women from late pregnancy –3^rd^ trimester (32–36 weeks) to 3 months postpartum n = 530 CPS = 223 PSG = 267 SOC = 263	Pregnant women were randomised to 3 groups A. Continuous cell phone based peer support (CPS) B. Monthly peer support group (PSG) C. Standard of care (SOC)	*Primary outcome:* Exclusive BF at 3 months	*BF initiated within 1 h:* CPS 73.0%, PSG 70.2%, SOC 67.2%, *P* = 0.519; OR for CPS vs SOC 1.32 (95% CI 0.82–2.12) *Onset of lactation >3days:* CPS 10.3%, PSG 8.9%, SOC 11.2%, *P* = 0.764; OR for CPS vs SOC 1.09 (95% CI 0.55–2.19) *Exclusive BF at 3 months:* CPS vs SOC: 90.9%vs 78.2% (Chi–square 9.8201, *P* = 0.0017) CPS vs PSG: 90.9%vs 82.8% (*P* = 0.032) OR for CPS vs SOC 2.77 (95% CI 1.44–5.32)	Low	Peer or group support (socially–mediated) – Continuous peer support (Cell phone)
Sharma et al. (2011), India [43]	RCT	Preschool children and their mothers Total N = 143 Intervention group = 71 Control group = 72	Oral health education via SMS vs pamphlet	*Primary outcomes:* Mothers’ knowledge, attitude, and practice of child’s oral health; Visible Plaque Index (VPI)	*Mean KAP scores for knowledge:* Pre–intervention: SMS group 8.2 ± 1.2; pamphlet group 7.8 ± 1.5 Post–intervention: SMS group 9.4 ± 0.8; pamphlet group 8.8 ± 1.1 Differences between groups: –0.43 (95% CI –0.33 to 0.51) *Mean KAP scores for attitude:* Pre–intervention: SMS group 8.8 ± 1.3; pamphlet group 7.8 ± 1.8 Post–intervention: SMS group 9.4 ± 0.7; pamphlet group 8.8 ± 1.3 Differences between groups: –0.37 (95% CI –0.61 to –0.13) *Mean KAP scores for practices:* Pre–intervention: SMS group 11.3 ± 1.8; pamphlet group 11.1 ± 1.8 Post–intervention: SMS group 12.1 ± 1.3; pamphlet group 11.5 ± 1.7 Differences between groups: –0.44 (95% CI –0.77 to –0.12) *Mean KAP scores for VPI:* Pre–intervention: SMS group 45.0 ± 21.2; pamphlet group 45.4 ± 20.5 Post–intervention: SMS group 33.5 ± 17.0; pamphlet group 35.6 ± 16.2 Differences between groups: 1.81 (95% CI –1.39 to 5.01)	High	Health Information Delivery – Health ‘education’/promotion (SMS)
Simonyan et al. (2013), Mali [44]	Quasi–experimental study, General population	0–72 months old with no diagnosed chronic diseases Total N = 188 Intervention group = 99 Control group = 89	Diagnosis, collection and transfer of health care data using mobile phone via a JAVA applet to a central server vs usual care	*Primary outcome:* Healthcare utilisation *Secondary outcomes:* Child morbidity indicated by number of episodes of cold, cough, diarrhoea, fever, infection, pain, teething, vomiting, wounds, and others	*Healthcare utilisation:* Mobile phone group 93.4%; control group 31.5%; OR 2.2 (95% CI 1.3–3.9) Total number of disease episodes: Mobile phone group 236; Control group 168 Episodes for specific disease are given in the paper. These were not statistically significantly different from the two groups.	High	Data collection (health monitoring or case finding by Community Health Workers)

ASR – adjusted standardized residuals, CPS – continuous cell phone based peer support, DS – Down Syndrome, EBF – effective breastfeeding, ICC – interclass correlation coefficient, IYCF – infant and young child feeding, NICU – Neonatal Intensive Care Unit, OR – odds ratio, aOR – adjusted odds ratio, PSG – peer support group, RCT – randomized controlled trial, RR – relative risk, SD – standard deviation, SES – socio–economic status, SMS – short message service, SOC – standard of care

* Only abstract available.

### Assessment of risk of bias

Risk of bias grading for the different components of each study is shown in Tables s3 and s4 in the **Online Supplementary Document(Online Supplementary Document)**. Only two of the intervention studies were graded as being at low risk of bias [36,42], seven as moderate [29,30,32,34,37–39] and four at high [28,33,43,44] risk of bias (see Table s3 in **Online Supplementary Document(Online Supplementary Document)**). One cohort study was graded high risk of bias [31], while a case–control and a before–and–after study were graded moderate risk of bias (see Table s4 in **Online Supplementary Document(Online Supplementary Document)**) [40,41]. Two of the studies included in our review were available only as conference abstracts [35,42]. Both sets of authors were contacted for further information and one replied, providing additional data that enabled us to better assess that study [42].

### Mobile delivery media

The delivery modes used were mobile phones with SMS (n = 11) [28,32–34,36–39,41–43], SMS and voice messaging (n = 1) [30] and voice calls (n = 2) [35,40]. Two studies used mobile applications to collect data [31,44] and one study used MP3 players to deliver audio recordings [29].

### Types of interventions

**Figure 2:**
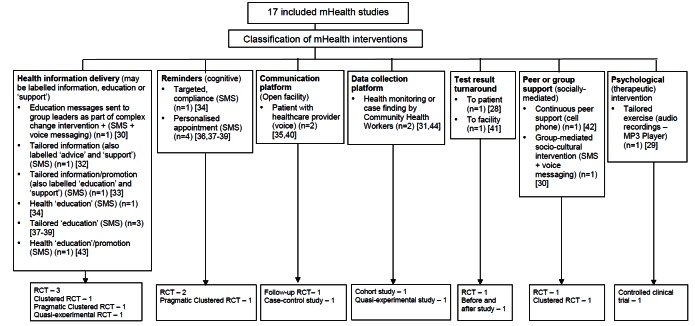
Classification of mHealth interventions of included studies. Categories are as interpreted by the reviewers, based on study descriptions. The authors may label studies somewhat differently. For example, the word ‘support’ may be used to describe informational messages, such as where it is theorized that these may confer psychological support in addition to knowledge support (eg, knowing that it is normal to experience morning sickness), although rarely do the authors elaborate on this. Studies are included in more than one category if the intervention is multi–faceted.

We classified the interventions according to our interpretation of their aims, based on the descriptions provided in the study reports, having first assessed existing taxonomies and found them to be not ideally suited to our purposes [20–22]. Studies were included in more than one category if the intervention was multi–faceted. The categories were health information delivery (n = 6) [30,32,33,34,37-39,43], reminders (n = 3) [34,36, 37-39], communication platform (n = 2) [35,40], data collection platform (n = 2) [31,44], test result turnaround (n = 2) [28,41], peer/group support (n = 2) [30,42], and psychological intervention (n = 1) [29]. The results of this classification exercise are shown in **Figure 2**.

### Types of outcomes examined

Eight studies examined indicators of maternal, newborn and child morbidity and mortality [28,29,31,32,34,37,40,44]. These covered maternal death [37], indicators of anaemia [34], duration of gestation at birth or preterm delivery [29,32], perinatal death and stillbirth [29,37], birth weight [30,31], Apgar score [28], hospitalization [29], route of delivery [29,31], infectious diseases [40,44], and oral health [43]. Other outcomes included indicators of infant feeding and breastfeeding [30,33,42], utilisation of antenatal, intrapartum, and postnatal care [31,35,37–40,44], quality of care [36,38], recording and collection of study data [31,40], indicators of self–efficacy [28,33], and compliance with recommended practices, such as micronutrient intake and uptake of immunization [34,36-38]. We did not find any study evaluating the cost–effectiveness of mHealth. The results are organised below according to the types of outcomes examined in each study.

### Effects on maternal, newborn and child morbidity and mortality

A Taiwanese CCT compared pregnancy outcomes in women at risk of pre–term labour who had received daily 13–minute relaxation therapy sessions delivered via mp3 player, as compared with routine prenatal care (**Table 1**) [29]. Women in the intervention group had longer pregnancies, but there was no difference in the rate of pre–term birth, birth weight, perinatal mortality or Apgar score.

In a RCT from Thailand [32], the duration of gestation, birth weight, preterm delivery and caesarean section were comparable in pregnant women receiving SMS prenatal support via mobile phone to those who received routine prenatal care (**Table 1**). Similar results were seen in a pragmatic cluster RCT from Zanzibar, Tanzania [37–39], in which women receiving SMS prenatal support were comparable to those who received routine prenatal care, however, the risk of perinatal death decreased by half in the SMS group compared to the routine care group (odds ratio (OR) 0.50, 95% confidence interval (CI) 0.27–0.93) (**Table 1**). An Iranian RCT evaluated a 12–week programme of SMS reminders encouraging compliance with iron supplementation. While self–reported compliance was greater in the intervention group than in a control group not receiving the SMS reminders, there was no difference between the groups in objective measures of serum iron [34] (**Table 1**).

A Nigerian case–control study [40] compared rates of facility utilization and maternal morbidity in health care facilities where pregnant women had received mobiles as a communication platform. No measurable differences were observed between the two samples (**Table 1**).

A quasi–experimental study from Mali of children aged 0–72 months [44] did not reveal differences in the incidence of childhood diseases between those whose health care data and diagnosis were recorded and transferred using mobile phone compared to children whose data were not recorded using mobile phone (**Table 1**).

### Effects on infant feeding

**Figure 3:**
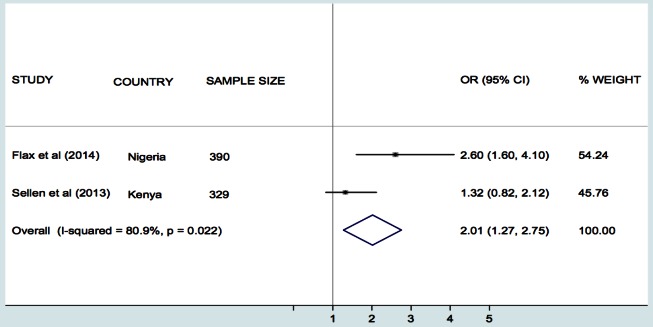
Meta–analysis of the effect of SMS/cell phone intervention vs routine prenatal care on initiation of breastfeeding within one hour after birth based on two RCT undertaken in Nigeria and Kenya: OR represents the odds ratio of effect.

**Figure 4:**
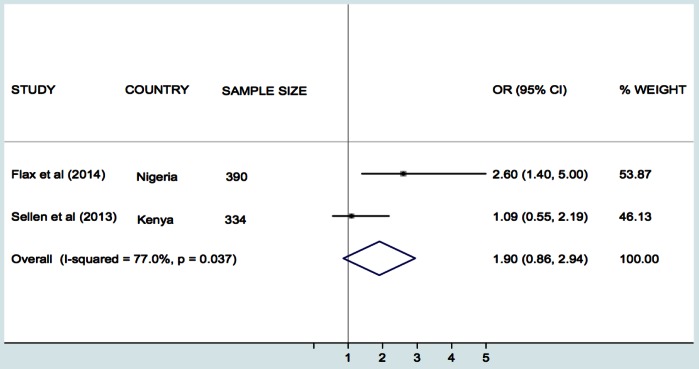
Meta–analysis of the effect of SMS/cell phone intervention vs routine prenatal care on onset of lactation within three days after birth based on two RCT undertaken in Nigeria and Kenya: OR represents the odds ratio of effect.

**Figure 5:**
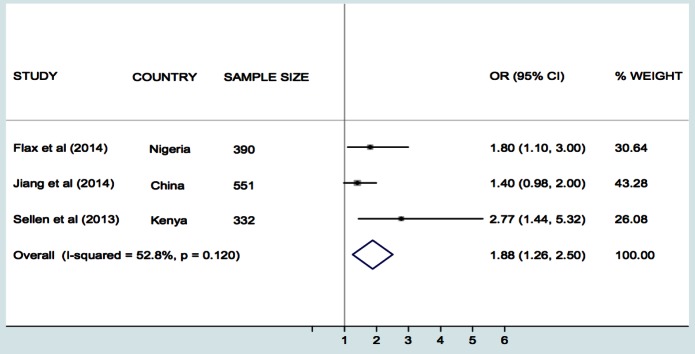
Meta–analysis of the effect of SMS/cell phone intervention vs routine prenatal care on exclusive breastfeeding for three or four months based on three RCT undertaken in Nigeria, China, and Kenya: OR represents the odds ratio of effect.

**Figure 6:**
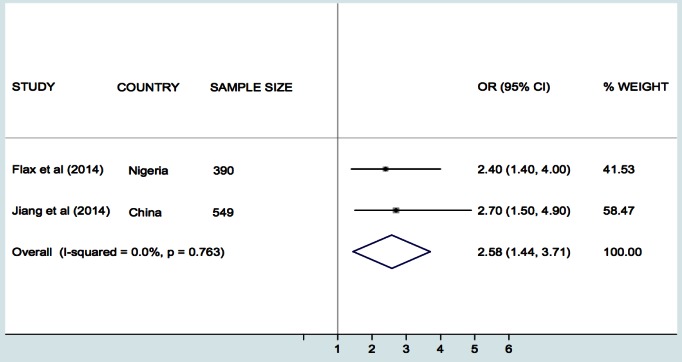
Meta–analysis of the effect of SMS/cell phone intervention vs routine prenatal care on exclusive breastfeeding for six months based on three RCT undertaken in Nigeria and China: OR represents the odds ratio of effect.

Flax et al. [30], Jiang et al. [33], and Sellen et al. [42], compared the effect of SMS/cell phone vs no SMS (routine prenatal care) on breastfeeding in Nigeria, China and Kenya, respectively. The results of each trial showed that the rate of exclusive breastfeeding (EBF) for three or four months was higher in the SMS/cell phone group than in the non–SMS/cell phone group (**Table 1**). We undertook meta–analyses of the effect of SMS/cell phone vs routine prenatal care on the initiation of breastfeeding within one hour after birth [30,42], giving colostrum or breast milk within three days after birth [30,42], and EBF at three/four months [30,33,42], and at six months [30,33]. The pooled estimates showed that the rates of initiating breastfeeding within one hour after birth (OR 2.01, 95% CI 1.27–2.75, I^2^ = 80.9%, **Figure 3**) were higher in the groups given a SMS/cell phone prenatal intervention than in groups not given the SMS/cell phone intervention. The evidence for giving colostrum or breast milk within three days after birth was not strong (OR 1.90, 95% CI 0.86–2.94, I^2^ = 77.0%, **Figure 4**). The rates of EBF for three/four months (OR 1.88, 95% CI 1.26–2.50, I^2^ = 52.8%, **Figure 5**) and EBF for six months (OR 2.58, 95% CI 1.44–3.71, I^2^ = 0.0%, **Figure 6**) were also higher in the groups given a SMS/cell phone prenatal intervention than in groups not given the SMS/cell phone intervention.

### Effect on *health care utilisation and quality of care*

In a follow–up study of a RCT in Bangladesh, Labrique et al. assessed the level of use of mobile phones by pregnant women in reporting obstetric emergencies [35]. 55% of pregnant women reported having used the mobile phones to obtain medical advice, call a health care provider, arrange for transportation or ask for financial support.

A Chinese RCT evaluated the effects of SMS–based appointment reminders for parents with children 0–18–years diagnosed with cataract and attending the paediatric clinic of a specialist eye hospital [36]. Attendance at follow–up clinics was higher in the group receiving SMS reminders than in those with standard appointments (91% vs 62%). This was associated with more surgeries, laser treatment for capsular opacification, prescription of new glasses and treatment for ocular hypertension in the intervention group, compared with those not receiving these reminders (**Table 1**). No subgroup analysis was reported for the under 5s.

Among pregnant Tanzanian (Zanzibar) women [37–39], those given mobile phones in order to receive SMS information about antenatal care were more likely to attend four or more antenatal care clinics (OR 2.39, 95% CI 1.03–5.55) and have skilled attendance at delivery (OR 5.73, 95% CI 1.51–21.81) than those who received routine prenatal care. No strong evidence of differences regarding tetanus vaccination, intermittent preventive treatment during pregnancy and antepartum referral were found (**Table 1**).

Healthcare utilisation was higher in pregnant Nigerian women from health facilities receiving mobile phones compared to women from health facilities without mobile phones (OR 1.32, 95% CI 1.15–1.53) [39].

Finally, Simonyan et al. found that health care utilisation was higher in Malian children whose health care data and diagnosis were collected and transferred using mobile phones compared with children whose data were collected and transferred using standard methods (OR 2.20, 95% CI 1.3–3.9) [44].

### Ongoing studies

Seven ongoing studies assessing the influence of mHealth interventions on maternal and child health outcomes in LMIC were identified in the course of the review. Three of these are being undertaken in Kenya, one each in Cameroon, Ethiopia, India and Mozambique. Six studies involve pregnant women and one involves children as participants. The mHealth interventions in all studies involve SMS or voice calls via mobile phones. (see Table s5 in **Online Supplementary Document(Online Supplementary Document)**)

## DISCUSSION

The current evidence base contains many studies describing the use of mHealth for supporting MNCH in LMIC but comparatively few have robustly evaluated the impacts of these interventions on health outcomes in these groups.

The majority of included studies took place in Sub–Saharan Africa and East Asia, while a few were undertaken in South Asia and the Middle East. Most studies were at moderate risk of bias. Although heterogeneity between studies precluded the calculation of a pooled estimate, mHealth interventions did not improve indicators of maternal, newborn, and child morbidity and mortality, except in one study from Tanzania that reported a decreased risk of perinatal death with use of SMS for prenatal support during pregnancy. However, a meta–analyses of three studies judged to be sufficiently homogenous showed that delivering prenatal breastfeeding interventions using SMS/cell phone (vs routine prenatal care) improved rates of initiation of BF within one hour after birth and increased the likelihood of EBF for up to six months, although there was no strong evidence regarding the giving of colostrum or breast milk within three days after birth.

mHealth technologies are increasingly being used to enhance health care utilisation, improve the quality of pre– and post–pregnancy care, and as a means of collecting pregnancy and child health data. Some studies showed that mHealth interventions, particularly those delivered using SMS, were associated with increased utilisation of health care, including uptake of recommended prenatal and postnatal care consultation, skilled birth attendance, and vaccination.

Most authors did not fully explain the basis of their intervention, in terms of its components or the mechanisms through which it would deliver the intended outcomes, and overall the studies lacked a common taxonomy for describing the type and purpose of the intervention. For example, the term ‘support’ was sometimes identified with health information delivery whereas elsewhere with a more psychosocial intervention. To aid interpretation and comparison we developed a framework for classifying the interventions according to their purpose, as previously described (see **Figure 2**). Based on our interpretation, the most common use of mHealth was for *health information delivery,* such as nutritional advice [30, 32, 33, 34, 37-39, 43]. This was followed by *reminders*, chiefly for clinic attendance [34,36, 7-39]. The other observed categories were mHealth as a *communication platform*, mainly to access support from care providers [35,40]; as a *data collection platform*, to enable birth registration or reporting of health indicators [31,44]; for accelerating *test result turnaround* times through by–passing the need for physical transportation [28,41]; part of *peer–*support [30,42]; and as a means by which to deliver *psychological (therapeutic)* interventions [29].

This systematic review draws on a comprehensive, inclusive and highly sensitive literature search strategy, analyses both health and health care utilization indicators; includes all legitimate mHealth technologies, covers the full spectrum of maternal and infant health and was not restricted by language. It has successfully captured the body of quantitative comparative studies on mHealth for MNCH through analysing a very large initial corpus of studies, and not simply those specified by the World Bank list of LMIC which, our pilot searches revealed, would have excluded key trials that we were aware of.

Comparable reviews have lacked such a robust search strategy [18], or have focused on the operational functions of mobile technologies rather than their outcomes [19,46]. In addition to those described in our introduction, new reviews arising after the publication of our protocol have similar limitations: Aranda–Jan and colleagues reviewed a range of mHealth studies carried out in Africa using only two databases [47], while Hall et al.’s review assessing ‘what interventions work’ for a range of conditions, was limited to two databases and grey literature [48]. As already noted, although Free et al.’s review covered a broad range of mHealth interventions, the majority of the trials revealed were from high income countries [20,21], whilst a systematic review on mHealth for LMIC, mentioned in Philbrick’s broader scoping review, is not available for comparison [17].

As with many systematic reviews in the field of eHealth, this analysis is limited by the difficulty of interpreting and synthesizing complex intervention studies and the variable description of interventions across studies. Although Labrique et al. developed a taxonomy for categorising different types of eHealth interventions [22], which we considered at the protocol stage, it did not fit our specific requirement to describe the interventions in terms of their purposes, for which the framework in Figure 2 was developed. Further work is needed to refine and test this with a larger body of interventions and to establish how best to integrate it with the various other published frameworks that exist. Due to the heterogeneity of the interventions and study outcomes we were unable to undertake meta–analyses, except in the case of the studies on infant feeding interventions, although this should be interpreted with caution due to the small number of studies analysed.

Our inclusion of studies from Taiwan is debatable, given its relatively high GDP but official status as part of China, which, although classified as ‘upper middle’ since 2012, is still a developing country. This, and our need to drop country restrictions from the search strategy due to labeling effects (eg, Zanzibar vs Tanzania), indicates taxonomic and socio–political challenges for systematic reviews of global research that warrant further methodological study.

Overall, the quality of studies included in the review was moderate, highlighting the importance of improving the methodological rigor of future research. For randomised trials, there is need for allocation concealment and adequate blinding of outcomes, while the quality of observational studies will be improved through prospective–designs and adjustment for confounding variables.

### Departure from protocol

World Bank Country Classification [25] was used instead of United Nations Human Development Index, due to our focus on income level rather than other aspects of development. The outcomes remain unchanged.

## CONCLUSIONS

There is a growing body of research indicating the potential of mHealth interventions for improving MNCH in LMIC, but overall the available evidence is weak and the results, in most cases, are too inconsistent to enable robust conclusions to be drawn about impacts on patient health outcomes. However supportive evidence exists with respect to the use of SMS/cell phones for improving infant feeding. Further research, using rigorous methodologies, is needed to better establish the effectiveness of mHealth interventions in MNCH initiatives in LMIC. In particular, trials with quantifiable economic, clinical and long–term patient–centred health outcomes are warranted. A number of in–progress trials are set to supplement this literature, while new research investments hold great promise for the development and evaluation of mHealth innovations for MNCH and other health priorities [49]. As low–cost smartphones begin to penetrate in these regions, a new generation of mobile Apps is now emerging, which will also require evidence–based methods to establish their safety, efficacy and societal impacts [15,50]. Innovative methods of integrating real–time evaluation into these deployments will also be essential if the potential evidence to be gained from them is to be effectively captured.

Our experience of engaging with this literature during the review also supports the common assertion that mHealth research projects are typically under–theorised, poorly specified and vaguely described. This creates challenges for effective evidence synthesis, risks unintended consequences that cannot be explained, makes replication and scaling difficult and hinders the effective translation of research to practice. We recommend that mHealth researchers, sponsors, and publishers prioritise the transparent reporting of interventions in terms of their aims, contexts, modes of delivery and presumed mechanisms of impact. Although anecdotal evidence of the benefits of mHealth for MNCH in LMIC is compelling, without this level of specification it will be difficult to develop robust evidence–based recommendations for policymakers and planners wishing to make informed choices about mHealth investments in these regions.
