# Supplementary material for: Effectiveness of mHealth interventions for maternal, newborn and child health in low– and middle–income countries: Systematic review and meta–analysis
Source: J Glob Health. 2015 Nov 8;6(1):010401. doi: 10.7189/jogh.06.010401 (PMC4643860; doi:10.7189/jogh.06.010401)
Supplement: Online Supplementary Document [file jogh-06-010401-s001.pdf]

Online Supplementary Document

Lee et al. Effectiveness of mHealth interventions for maternal, newborn and child health in low– and middle–income countries: Systematic review and meta–analysis

JoGH 2016;6:010401

Table s1. Search strategy: Medline format

1. (eHealth or e-Health).mp.
2. (mHealth or m-health or mobile health).mp.
3. Telemedicine/ or (telecare or telehealthcare or mobile telehealthcare or mobile telemedicine or mCare or m-care).mp.
4. apps or mobile applications/
5. (mobile communication or mobile technology or mobile devic*).mp.
6. Computers, Handheld/ or Microcomputers/ or (tablet computers or mobile tablet computers or mobile technolog*).mp.
7. Communication satellite.mp.
8. Cellular phone/ or (cellular phone* or cell phone or mobile phone).mp.
9. MP3 player*.mp.
10. Text Messaging/ or (texting or text messag* or messag* or text* or short message or SMS or multimedia technol* or multimedia messag* or multi-media messag).mp.
11. (Personal digital assistant* or PDA).mp.
12. (Smartphone or smart-phone).mp.
13. (podcast* or pod-cast*).mp.
14. Social media/ or Social networking/ or (Twitter or Facebook).mp.
15. (Global positioning system or GPS).mp.
16. Radio Frequency Identification Device/ or RFID.mp.
17. or/1-16
18. Pregnancy/ or Pregnant women/ or Pregnancy outcome/
19. Parturition/ or childbirth.mp.
20. Obstetrics/
21. Maternal Health Services/ or matern*
22. (pregnan* or maternal or maternal health).mp.
23. Labor, or Labour or Obstetric/
24. Delivery, Obstetric/
25. Midwifery/ or Traditional Birth Attendant.mp.
26. Postpartum period/ or puerperium.mp.
27. Delayed delivery.mp. or three delays.mp.

28. Pregnancy complications/ or Obstetric Labor complications/ or Obstetric Labor, Premature/ or Puerperal Disorders/ or Depression, Postpartum/ or Maternal Mortality/
29. Infant, Newborn/ or neonat*.mp.
30. Prenatal Care/ or Perinatal Care/ or Postnatal Care.mp.
31. (Antenatal care or intrapartum care or postpartum care or post-partum care or puerperal care).mp.
32. (Perinatal complication* or postnatal complication*).mp.
33. Birth injuries/
34. Breastfeeding/ or (breastfeeding or breast-feeding).mp.
35. or/18-34
36. Intervention studies/ or experimental studies.mp.
37. analytical stud*.mp.
38. Clinical trial/ or Controlled Clinical Trial/ or Randomized Controlled Trial/ or (clinical trial or controlled clinical trial or randomi?ed controlled trial).mp.
39. Double-Blind Method/ or Single-Blind Method/ or (double-blind design or single-blind design).mp.
40. Placebos/ or Random Allocation/ or random*.mp.
41. (Controlled before and after stud*).mp.
42. Interrupted time series.mp.
43. Cohort studies/ or (cohort stud* or cohort).mp.
44. (control or healthy control).mp.
45. Case-control studies/ or case-control stud*.mp.
46. or/36-45
47. 17 and 35 and 46
48. limit 47 to year="1990-2014"
49. limit 48 to human

Table s2. Search strategy: free-field format

(mHealth or m-Health or eHealthor e-Health or telemedicine or mobile health or mobile telehealthcare or mobile phone or cellular phone or personal digital assistant or mobile tablet computers or smart phone or mobile technology or apps or mobile applications or text messag* or short messag* or SMS or multimedia messag*)
AND
(child* or infant* or baby or babies or neonatal or newborn* or preterm* or prematur* or pregnan* or pregnant women or mother* or obstetric labor or obstetric labour or obstetric delivery or obstetric labor complications or midwifery or traditional birth attendant or perinatal care or prenatal care or antenatal care or intrapartum care or postnatal care or perinatal complications or postnatal complications)
AND
(analytical stud* or epidemiologic* or compar* or evaluat* or follow-up or follow-up or prospective or retrospective observation* or cohort or case-control or trial* or RCT or controlled before and after study or interrupted time series or intervention* or prospective or retrospective or control* or double-blind or single-blind or random*)

Table s3. Risk of bias assessment of included intervention studies based on the criteria of the Cochrane Handbook for Systematic Reviews of Interventions

Study, year	Study Design	Adequate sequence generation	Allocation concealment 1.	Blinding/ patient-related outcomes	Incomplete outcome data addressed?	Free of selective reporting	Free of other bias	Overall Grade
Cheng et al, 2008	RCT	Unclear	Unclear	Unclear	No	Yes	Unclear	High risk
Chuang et al, 2012	CCT	Yes	Yes	Yes	Yes	Yes	Unclear	Moderate risk
Flax et al, 2014	RCT	Yes	Yes	No	Yes	Yes	No	Moderate risk
Jareethum et al, 2008	RCT	Yes	Unclear	Unclear	Yes	Yes	Yes	Moderate risk
Jiang et al, 2014	Quasi-RCT	Unclear	No	Yes	Yes	Yes	No	High risk
Khorshid et al, 2014	RCT	Yes	No	Unclear	Yes	Yes	Yes	Moderate risk
Lin et al, 2012	RCT	Yes	Yes	Yes	Yes	Yes	Yes	Low risk
Lund et al, 2012, 2014a, 2014b	RCT	Yes	Unclear	Unclear	Unclear	Yes	Yes	Moderate risk
Sellen et al, 2013*	RCT	Yes	Yes	Yes	Yes	Yes	Yes	Low risk
Sharma et al, 2011	RCT	No	No	Unclear	No	Yes	No	High risk
Simonyan et al, 2013	Quasi-RCT	No	No	No	Unclear	Unclear	Unclear	High risk

Abbreviations:
*****Abstract
CCT - controlled clinical trial
RCT - randomised controlled trial

Table s4. Risk of bias assessment of included observational studies using the Effective Public Health Practice Project quality assessment tool

Study, year	Study Design	Selection Bias			Study Design			Confounders			Blinding			Data Collection Method			Withdrawals and Dropouts			Global Rating		
		S	M	W	S	M	W	S	M	W	S	M	W	S	M	W	S	M	W	S	M	W
Gisore et al 2012	Cohort	√				√				√			√	√			√					√
Oyeyemi et al 2014	Case-control		√			√				√			√		√		√				√	
Seidenberg et al 2012	Uncontrolled before and after study		√			√				√			√		√			√			√	

EPHPP – the Effective Public Health Practice Project,

S – Strong,

M – Moderate,

W – Weak

Table S5. Characteristics of on-going studies

Author/ Country	Title of study	Study design	ClinicalTrials.gov identifier	Population (N)/ Sample size (n)	Intervention/ Exposure	Outcomes	Estimated date for reporting final results	Notes
Cesar de Palha et al. Mozambique	Evaluation of the impact of mobile phone messages on ART and PMTCT adherence in Mozambique: a randomized control trial	RCT	NCT01910493	1352 pregnant women, older than 18 years between 8-28 weeks of gestation	Behavioural: SMS reminders	*Primary outcome*<br>Retention in ART care<br><br>*Secondary outcome*<br>Retention in PMTCT care	This study has been completed.	University of Eduardo Mondlane: Mozambique Ministry of Health: Mozambique
Kimani J et al. Kenya	Harnessing Mobile Phone Usage for HIV and Horizontal Health Systems Improvement: PMTCT	RCT	NCT01157442	856 pregnant women from 18 to 49 years with singleton pregnancy	Behavioural: cell phone SMS text messaging	*Primary outcome:*<br>Increased nevirapine uptake in labour in pregnant HIV positive women from 60% to 70%<br><br>*Secondary outcome:*<br>HIV positive rates in infants born to mothers in the study; number of antenatal care visits; earlier identification and treatment of HIV positive infants; acceptability of cell phone SMS text messages for PMTCT related care	This study is currently recruiting participants.	jkimani@csrtkenya.org
Lund S et al. Ethiopia	Evaluation of a Smartphone Application to Reduce Adverse Pregnancy Outcomes in Ethiopia: A Cluster-	RCT	NCT01945931	4770 10 to 45 years pregnant women in active labour	Safe Delivery Smartphone Application	*Primary outcomes:*<br>Perinatal mortality (stillbirth and early neonatal death);<br>Postpartum haemorrhage<br><br>*Secondary outcomes:*<br>Health workers clinical performance in active management of third stage of labour, postpartum haemorrhage	This study is currently recruiting participants.	stine_lund@dadlnet.dk

	randomised Controlled Trial					and manual removal of placenta;<br>Health workers clinical performance in management of neonatal resuscitation;<br>Health workers knowledge of management of active management of third stage of labour, manual removal of placenta and postpartum haemorrhage;<br>Health workers knowledge of management of active management of third stage of labour, manual removal of placenta and postpartum haemorrhage		
Mazumder S et al.<br>India	mHealth – a novel approach to improve child nutrition in India	Interven tional	NCT01645163	72 children aged 9-11 months	Mobile phone counselling – feasibility & acceptability of using mobile phones to improve complementary feeding practices.	*Primary outcomes:*<br>mHealth to improve complementary feeding – before and after counselling.	Recruiting Estimated completion: August 2012	Contact: chrd@sas.org.in
Mutarambirwa PC et al.<br>Cameroon	Using mobile phones to promote utilisation of reproductive healthcare services. The Lagdom Health pilot study	RCT	PACTR201210000424220	430 pregnant women aged 16 years and above, all stage of pregnancy	Use of calls, SMS and voicemails. Investigate the efficacy of mobile phones in increasing antenatal care service utilisation and reducing delays of pregnant women in distress in accessing	*Primary outcomes:*<br>Antenatal clinic attendance rate.<br><br>Time to see a skilled birth attendant.	Active recruitment	Contact: adra_cameroon@yahoo.fr

					emergency obstetric care services.			
Ong'ech J et al.<br>Kenya	Mobile Phone Technology for Prevention of Mother-to-Child Transmission of HIV: Acceptability, Effectiveness, and Cost	RCT	NCT01645865	600 HIV-positive pregnant women up to 32 week of gestation seeking ANC at a study site	Behavioural	*Primary outcomes:*<br>The proportion of women who successfully complete key PMTCT transition points from antenatal to six weeks postpartum;<br>Initiation of Infant prophylaxis, Facility delivery and receipt of results of 6 weeks early infant diagnosis by DNA PCR<br><br>*Secondary outcomes:*<br>Uptake ARV prophylaxis/ART during labour, delivery, and postpartum;<br>Self-reported maternal adherence to ARV prophylaxis/ART during pregnancy;<br>Time to initiation of ARV prophylaxis/ART uptake after initial identification of HIV seropositivity within ANC	Time to initiation of ARV prophylaxis/ART uptake after initial identification of HIV seropositivity within ANC<br>April 2014	Elizabeth Glaser Pediatric AIDS Foundation,
Unger J et al.<br>Kenya	Mobile Phone One Way Short Message Service (SMS) Versus SMS Dialogue for Women's and Child Health (Mobile WaCH) in Kenya: A Randomized Control Trial	RCT	NCT01894126	300 14 years and older pregnant women	Behavioural: Two-way SMS Dialogue; One-way SMS Messaging	*Primary outcomes:*<br>Contraceptive uptake;<br>Facility Delivery<br><br>*Secondary outcomes:*<br>ANC attendance; Exclusive Breastfeeding; Infant Immunizations; Maternal morbidity; Infant morbidity	This study is currently recruiting participants.<br>May 2015	junger@uw.edu

Abbreviations:

AIDS – acquired immune deficiency syndrome

ANC – antenatal care
ARV – antiretroviral
DNA PCR – DNA polymerase chain reaction
PMTCT – prevention of mother to child transmission of HIV
RCT – randomized controlled trial
SMS – short message service
